# *KCNJ11* rs5219 Gene Polymorphism Is Associated With T2DM in a Population of Bangladesh: A Case-Control Study

**DOI:** 10.1155/ije/5834412

**Published:** 2025-05-02

**Authors:** Amrita Bhowmik, Begum Rokeya, Yearul Kabir

**Affiliations:** ^1^Department of Applied Laboratory Sciences, Bangladesh University of Health Sciences, Dhaka, Bangladesh; ^2^Department of Biochemistry and Molecular Biology, University of Dhaka, Dhaka, Bangladesh; ^3^Department of Pharmacology, Bangladesh University of Health Sciences, Dhaka, Bangladesh

**Keywords:** *KCNJ11*, polymorphism, RFLP, rs5219, T2DM

## Abstract

**Introduction:** As a polygenic disorder, Type 2 diabetes mellitus is a prevalent disease developed by many multigenetic factors, of which multiple genes located on different chromosomes contribute to its susceptibility. The *KCNJ11* gene is involved in the Kir6.2 proteins that help release insulin from the potassium channels in pancreatic beta cells. Many studies have found that *KCNJ11* polymorphism is significantly associated with the incidence of T2DM. Therefore, this study was carried out to investigate the association between *KCNJ11* gene polymorphism and T2DM in the Bangladeshi population.

**Materials and Methods:** In a case-control study (*n* = 697), 326 nondiabetic controls and 371 diabetic subjects (diagnosed based on American Diabetes Association criteria) were recruited for this study. The serum fasting glucose, lipid profiles, creatinine, alanine aminotransferase (ALT), HbA_1C_, and serum insulin level were measured by standard methods. HOMA-B%, HOMA-S%, and HOMA-IR were calculated using HOMA-SIGMA software Version 2.2. A standard formula calculated QUICKI and Secretory-HOMA. The chemical method was used for DNA extraction from whole blood samples. The PCR–RFLP method was used to detect *KCNJ11* polymorphisms by restriction enzyme (BanII) digestion. As appropriate, data were analyzed using an independent *t*-test, chi-square, or Fisher exact test. *p* < 0.05 was considered significant.

**Results:** The frequency of the risk allele K was significantly higher in the T2DM group than control (*p* ≤ 0.01). The frequency of the KK genotype was higher among the T2DM group (3.77% vs. 1.84%, *p* < 0.05), and the frequency of the EK genotype was significantly higher among the T2DM than the control group (42.86% vs. 27.91%, *p* < 0.001). The EE genotype was significantly associated with T2DM in the dominant model EE + EK with an OR of 2.06 (95% CI 1.51–2.82, *p* ≤ 0.001).

**Conclusion:** This study showed that rs5219 polymorphism of the *KCNJ11* gene is a significant risk factor for Type 2 diabetes mellitus in the Bangladeshi population.

## 1. Introduction

The incidence of diabetes dramatically increased over the past 2 decades and reached 537 million adults, predicted to rise to 643 million by 2030 and 783 million by 2045 in low- and middle-income countries [1]. The diabetic prevalence in Bangladesh has increased substantially in the adult population. A meta-analysis showed that it is from 4% in 1995–2000 to 5% in 2001–2005 to 9% in 2006–2010, respectively [2] and will be suspected to reach 13% by 2030 according to the International Diabetes Federation [3–5]. Type 2 diabetes mellitus (T2DM) is a single-nucleotide polymorphism (SNP) dominant polygenic disorder that develops through the complex interaction of multiple genes [6]. Among them, some are potassium voltage–gated channel subfamily J member 11–associated candidate genes that have significant attention for T2DM risk for their functional regulation of glucose-induced insulin secretion [7]. *KCNJ11* gene (11p15.1, exon one) encodes Kir6.2 proteins and forms the inner section of adenosine triphosphate–sensitive potassium (KATP) ion channel in pancreatic beta cells for insulin secretion. The substitution of glutamic acid in lysine amino acid at “adenine codon 23” shows overactivity of the K channel with inhibition of glucose-induced insulin secretion and reduction of potassium channels' sensitivity [8, 9]. Several SNPs of the *KCNJ11* gene have been detected; among them, rs5219 has been receiving more attention for its association with diabetes. However, there are inconsistent results in previous studies in Asian populations [9, 10]. Therefore, this study investigated the association of *KCNJ11* rs5219 polymorphism with T2DM in the Bangladeshi population.

## 2. Materials and Methods

### 2.1. Study Subjects

T2DM is a chronic metabolic disorder characterized by insulin resistance and relative insulin deficiency and has a strong genetic predisposition. About 75 independent genetic loci have been identified in the progression of diabetes [6]. In this study, about 697 Bangladeshi subjects were randomly selected for this study, with 371 Type 2 diabetics and 326 nondiabetic subjects as control. Subjects with T2DM were selected from the OPD; Bangladesh Institute of Research and Rehabilitation in Diabetes, Endocrine and Metabolic Disorders (BIRDEM); and Bangladesh Institute of Health Sciences Hospital (BIHSH) and nondiabetic control was selected from Bangladesh University of Health Sciences (BUHS) and University of Dhaka. Subjects have explained the study's objectives, and written informed consent was obtained by all participants. A questionnaire was prepared, including physical measurements (anthropometry, blood pressure profile, and body fat percentage), family history of diabetes, and clinical information of the participants. The case-control study was approved by the Institutional Ethical Review Committee (BMBDU-ERC/EC/17/09) of the Department of Biochemistry and Molecular Biology, University of Dhaka, and conducted following the Declaration of Helsinki and its subsequent revisions [11].

### 2.2. Collection of Sample

About 8.0 mL of blood was collected from each subject, of which, 3.0 mL was transferred to an EDTA tube and 5.0 mL to a vacutainer tube, from which serum was collected after being centrifuged at 3000 rpm for 10 min. Tubes were stored at −20°C until further use.

### 2.3. Assay of Biochemical Parameters

The serum fasting glucose, HbA_1C_, triglycerides, total cholesterol, HDL–C, LDL–C, creatinine, and alanine aminotransferase (ALT) levels were measured by standard laboratory methods, respectively. Serum fasting insulin level was measured by the enzyme-linked immunosorbent assay (ELISA) method. HOMA-B%, HOMA-S%, and HOMA-IR were calculated by HOMA-SIGMA software Version 2.2. A standard formula calculated QUICKI and Secretory-HOMA.

### 2.4. *KCNJ11* Genotyping

**Figure 1:**
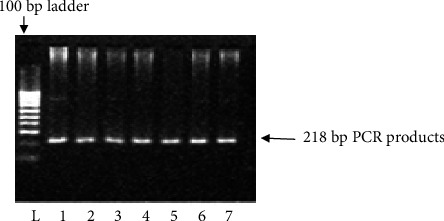
Representative PCR products of the *KCNJ11* gene in 3% agarose gel. Lanes 1–7: the presence of the PCR product of 218 bp and Lane L: the 100 bp ladder.

**Figure 2:**
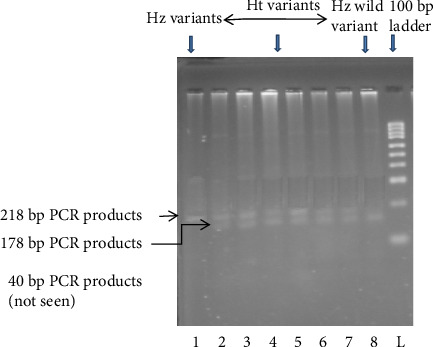
Representative digested PCR product of the *KCNJ11* (E23K) gene. L: 100 bp DNA ladder, Lane 1: homozygous (Hz) variants 218 bp, and Lanes 2–7: heterozygous (Ht) variants 218, 178, and 40 bp. Lane 8: complete digestion with 178 and 40 bp. The 40 bp fragment was passing out from the gel.

*KCNJ11* genotyping was performed using the PCR–RFLP method. Using the primers (forward: 5′-GAATACGTCCTGACACGCCT-3′; reverse: 5′-GCCAGCTGCACAGGAAGGACAT-3′), PCR amplified the genomic DNA. PCR primer was constructed according to the protocol of Ezenwaka et al. [12]. The primer sequences were verified using NCBI BLAST. After completion of PCR, an enzyme was used to digest the PCR products: Eco 241 (BanII) at 37°C for 16 h. The digested materials were separated by electrophoresis on a 3% agarose gel. Gels were stained with ethidium bromide, and electrophoretic bands were observed and captured on camera under ultraviolet light. Only a single band of 218 bp indicated that both polymorphic alleles were wild-type (E23E). Incomplete digestion products of 218, 178, and 40 bp indicated heterozygote (Ht) mutant allele (E23K), and completed digestion products of 178 and 40 bp indicated homozygote (Hz) mutant allele (K23K), respectively (Figures 1 and 2).

### 2.5. Statistical Data Analysis

All demographic and biochemical variables were studied and compared using an independent sample *t*-test. Genotype and allele frequencies, including risk factors, were compared in cases and control subjects using the chi-square test by GraphPad Prism, Version 7. Logistic regression analysis was used to calculate odds ratios (ORs) and 95% confidence intervals (CIs) to measure the relative risk after adjusting for gender, BMI, glucose, insulin, HOMA-B%, HOMA-S%, HOMA-IR, triglycerides, total cholesterol, and HDL cholesterol by using Statistical Package for the Social Sciences (SPSS), Version 24. To estimate the association of various clinical factors and diabetes, multinomial logistic regression models were performed for gender, BMI, glucose, insulin, HOMA-B%, HOMA-S%, HOMA-IR, triglycerides, total cholesterol and HDL–C as independent variables with E23K and total mutant E23K + K23K variants of *KCNJ11* genotype as dependent factors. Differences were considered significant at *p* < 0.05.

## 3. Results

### 3.1. Basic Characteristics of the Study Subjects

A significant difference was found between the ages of the two groups (*p* < 0.001). Females were higher in subjects with T2DM than in control, 53% and 47%, respectively. There was no difference in BMI value between case and control groups. Waist–hip ratio (WHR) shows the significant differences between the two groups. A significant (*p* < 0.001) difference in blood pressure (*p* < 0.001) and body fat percentage (*p* < 0.05) was found between subjects with T2DM and control, respectively. The positive family history of diabetes was significantly higher in subjects with T2DM compared to control (Table 1).

### 3.2. Clinical and Biochemical Data

The glucose, HbA_1C_, and insulin levels of subjects with T2DM were significantly higher (*p* < 0.001) than those of control subjects. On the other hand, the HOMA-B%, HOMA-S%, QUICKI, and Secretory-HOMA were significantly lower in subjects with T2DM compared to the control. HOMA-IR, triglycerides, and LDL levels were significantly higher, whereas HDL–C levels were significantly lower in subjects with T2DM compared to the control. No significant differences were found in ALT and creatinine levels between the two groups (Table 2).

### 3.3. Frequency Distribution of the *KCNJ11* Genotype and Risk of Diabetes

As shown in Table 3, 53.37% of patients with T2DM contained E23E, 42.86% contained E23K, and 3.77% contained K23K genotypes. Meanwhile, 70.25% of the control subjects contained E23E, 27.91% contained E23K, and 1.84% had K23K genotypes, respectively. Significant associations of genotype were found for E23K (*p* < 0.001), K23K (*p* < 0.05), and E23K + K23K (*p* < 0.001) in subjects with T2DM, when the E23E genotype was considered as the reference group. *KCNJ11* variants of E and K allele frequency were 0.748 and 0.252 in subjects with T2DM and 0.842 and 0.158 in control, respectively. K allele was significantly higher (*p* < 0.01) in subjects with T2DM than in control (Table 3).

### 3.4. Frequency Distribution of the *KCNJ11* Genotype and Risk of Diabetes According to Gender

A significant difference in the genotypic distribution of E23K was found in both diabetic males (*p* < 0.05) and females (*p* < 0.001) compared to the subjects with control when the E23E genotype was considered as the reference group. The result indicated that E23K variants might be risk factors for male and female subjects with diabetes (Table 4).

### 3.5. Frequency Distribution of the *KCNJ11* Genotype and Risk of Diabetes According to the Family History of Diabetes

The frequency of E23K variants of the *KCNJ11* genotypes was significantly higher in subjects with T2DM having a positive family history of diabetes compared to control. No association was found in subjects with T2DM and controls without a family history of diabetes. The result showed that the E23K genotype of *KCNJ11* may be a high risk for subjects with T2DM who have a positive family history of diabetes in comparison to those without a family history of diabetes (Table 5).

### 3.6. Distributions of Glycemic, Insulinemic, and Lipidemic Status According to the *KCNJ11* Genotype in the Study Subjects

Nonsignificant variations were found in fasting glucose (*p* = 0.526), HbA_1C_% (*p* = 0.601), and insulin (*p* = 0.811) among E23E, E23K, and K23K genotypes on subjects with T2DM, respectively. Serum triglycerides (*p* = 0.131), total cholesterol (*p* = 0.654), and LDL–cholesterol (*p* = 0.320) levels were also nonsignificantly higher, and HDL–cholesterol (*p* = 0.080) level was nonsignificantly lower in the Hz genotype (K23K) compared to E23E and E23K genotypes found in this study (data not shown).

### 3.7. Multinomial Logistic Regression Analysis for Risk Factors of Diabetes With the *KCNJ11* Genotype in Subjects With T2DM

The multinomial logistic analyses indicated that BMI (*p* < 0.01), glucose (*p* < 0.001), triglycerides (*p* < 0.05), total cholesterol (*p* < 0.01), and HDL–cholesterol (*p* < 0.001) were significantly associated with diabetes, whereas E23K variants showed a 1.76-fold risk factor for the incidence of diabetes (Table 6). This analysis also demonstrated that the genetic variant of *KCNJ11* was nonsignificantly different between the case and control after adjustment for confounding factors.

## 4. Discussions

Several studies reported a significant association of *KCNJ11* E23K with the incidence of T2DM [13–16]. Li noted that the *KCNJ11* E23K gene polymorphism is associated with T2D risk in the Chinese Han population [13]. In Iranian patients, Rabbani et al. also reported that *KCNJ11* (E23K) gene polymorphism is associated with T2DM. They found that the carrier Hz for the KK genotype is susceptible to T2D, and in patients, the frequency of the K allele was higher than in control subjects [14]. In India, the association between rs5219 and T2DM has also been reported [15, 17]. A previous meta-analysis study also established that rs5210 polymorphism was associated with T2DM [16, 18]. Wang et al. [9] reported that rs5219 K allele was relevant to T2DM risk in Caucasian and East Asian; the recessive genetic model indicated that the KK genotype was related to T2DM risk in Caucasian, East Asian, South Asian, and North African; and the dominant genetic model pointed out that the EE genotype was an opposite association with T2DM risk in Caucasian. However, in contrast, no association was established between rs5219 and T2DM in two case-control studies, the Khatri Sikh cohort and the North-Eastern people of India [19, 20]. Both studies were low-powered due to the small sample size. A meta-analysis in a new Dutch case-control study also reported no association of *KCNJ11* genotypes with diabetes [21]. Keshavarz et al. [10] also reported no associations of *KCNJ11* with T2DM in the population of Iran. It was found from our study that the frequency of Ht E23K (*p* < 0.001) and Hz K23K (*p* < 0.05) genotypes were significantly higher in subjects with T2DM compared to control when Hz E23E was considered as the reference group, which indicated a higher risk of diabetes for both variants. T2DM also shows that the carrier of the K allele was significantly higher (*p* < 0.01) in subjects with T2DM compared to the E allele, which demonstrated that the genotypic distribution of *KCNJ11* E23K polymorphism is highly associated with T2DM (Table 3).

In this study, a significant association of E23K genotypes with diabetes was found in both male (*p* < 0.05) and female (*p* < 0.001) subjects with T2DM, which indicated that the E23K genotype carries a higher risk for male and female subjects with T2DM (Table 4). No genderwise association with genotype was reported in other studies. Furthermore, the frequency of E23K variants was significantly higher (*p* < 0.001) in subjects with T2DM with a positive family history of diabetes (Table 5). Similarly, Sunita et al. [22] observed that subjects with a positive family history of T2DM possess a 6.66 times higher risk of having E23K and K23K genotypes than controls, and the risk of having the K allele in cases is 3.38 times higher than controls. In this study, no association was found in T2DM *KCNJ11* polymorphisms with glucose, HbA_1C_, insulin, and lipid profiles with *KCNJ11* genotypes. Hansen et al. [23] also reported that in the *KCNJ11* E23K variant, no significant difference in insulin secretion was observed for wild-type or Ht and Hz forms. However, in both variants, insulin secretion was decreased. Pietrzak-Nowacka et al. [24] also did not find any association of genotypes with glucose, insulin, TG, TC, HDL–C, and LDL–C.

On the other hand, Lasram et al. [25] reported an association between the E23K variant and various T2DM–related quantitative traits (fasting plasma glucose, BMI, TG, TC, HDL–C, and LDL–C, SBP, and DBP) among the T2DM, control group, and the overall sample. Fasting plasma glucose levels were higher in subjects with the EK genotype than in those with the KK and EE genotypes in the T2DM. When considering the overall sample, a different tendency was observed, and fasting plasma glucose levels were higher in subjects with the KK genotype than in those with the EK and EE genotypes. However, no significant differences were observed between genotypes and groups for BMI, triglycerides, total cholesterol, HDL–C, SBP, and DBP.

By multinomial logistic analyses, we found that BMI (*p* < 0.01), glucose (*p* < 0.001), TG (*p* < 0.05), TC (*p* < 0.01), and HDL–C (*p* < 0.001) in the *KCNJ11* (E23K) genotype showed a 1.76-fold higher diabetic risk (Table 6). Lasram et al. [25] reported a significant association between the E23K variant and T2DM after adjusting for age, gender, and BMI. Phani et al. [26] studied the susceptibility of T2DM–related quantitative traits (HbA_1C_, FPG, TC, TG, HDL–C, LDL–C, age at diagnosis, and BMI) on T2DM using the univariate general linear regression model for adjusting age, sex, and BMI as covariates. They showed that the *KCNJ11* rs5219 T/T genotype increased BMI and an early age of disease onset in subjects with T2DM. Several functional studies have identified an association between the rs5219 (E23K) *KCNJ11* polymorphism and an increased risk of T2DM as well as impaired insulin secretion [27–29].

In conclusion, our findings indicated that a common variant E23K in the *KCNJ11* gene was significantly associated with T2DM and may be utilized as a marker for the predisposition of T2DM as an aspect of Bangladesh. However, investigations with a larger sample size are required to confirm the findings that *KCNJ11* gene polymorphisms are responsible for the development of T2DM.

### 4.1. Limitations

The study was unable to implement next-generation sequencing (NGS) analysis due to the institution's lack of NGS facilities. We did not perform “random sample sequencing” for sample validation or incorporate additional genetic markers to provide a more comprehensive genetic risk profile of T2DM, or conduct “functional studies” to investigate the impact of the E23K variant on *KCNJ11* protein function and insulin secretion. Time and financial constraints also limited our ability to investigate other genes in this study. We acknowledge that replicating our study in independent cohorts from different regions of Bangladesh or neighboring countries would strengthen the validity and generalizability of our findings. However, due to limited resources and logistical challenges in sample collection, we were unable to conduct regional sampling. While gene–environment interactions were beyond the scope of this study, we recognize their significance and may explore them in future research. Conducting longitudinal studies would provide valuable insights into the long-term impact of *KCNJ11* polymorphisms on T2DM progression and prognosis. Although the sample size was adequate, it may limit the generalizability of the results to broader populations. Future research should address these limitations by incorporating larger sample sizes, functional validation studies, and longitudinal data.

Despite these constraints, this study makes a valuable contribution to understanding KCNJ11 and its implications for T2DM, providing a foundation for future investigations and potential clinical applications.

## Figures and Tables

**Table 1:** Basic characteristics of the study subjects.

Variables	Study subjects		*p* value
	Diabetic (*n* = 371)	Control (*n* = 326)	
^*#*^ *Gender*			
Male	177 (47)	172 (53)	—
Female	194 (53)	154 (47)	—
Age (year)	49 ± 10	37 ± 9	0.001
BMI (kg/m^2^)	25.3 ± 3.8	25.3 ± 3.8	0.539
Waist–Hip ratio	0.9 ± 0.2	0.9 ± 0.1	< 0.001
SBP (mmHg)	121 ± 12	112 ± 14	< 0.001
DBP (mmHg)	79 ± 7	75 ± 9	< 0.001
Body fat (%)	31 ± 8	29 ± 6	< 0.05

^*#*^ *Family history of diabetes*			
No	113 (30.5)	194 (59.5)	0.001
Yes	258 (69.5)	132 (40.5)	

*Note:* Results are presented as mean ± standard deviation (SD).

Abbreviation: ns = not significant.

^#^Numbers (percentages).

**Table 2:** Clinical and biochemical data of the study subjects.

Variables	Study subjects		*p* value
	Diabetic (*n* = 371)	Control (*n* = 326)	
Glucose (mM/L)	7.5 ± 2.5	4.9 ± 0.6	< 0.001
HbA_1C_ (%)	7.8 ± 1.7	5.1 ± 0.5	< 0.001
Insulin (μU/L)	23.8 ± 14.2	14.5 ± 5.8	< 0.001
GINR	0.41 ± 0.29	0.44 ± 0.32	0.211
HOMA-B%	117.7 ± 79.7	149.0 ± 55.9	< 0.001
HOMA-S%	41.5 ± 24.8	69.3 ± 46.7	< 0.001
HOMA-IR	3.2 ± 1.8	1.8 ± 0.7	< 0.001
QUICKI	0.29 ± 0.03	0.32 ± 0.03	< 0.001
Secretory-HOMA	208.9 ± 184.3	263.2 ± 180.8	< 0.01
Triglycerides (mg/dL)	152 ± 80	133 ± 47	< 0.01
Total cholesterol (mg/dL)	166 ± 53	171 ± 33	0.116
HDL–cholesterol (mg/dL)	40 ± 11	51 ± 14	< 0.001
LDL–cholesterol (mg/dL)	108 ± 47	95 ± 33	< 0.001
TC: HDL–C	3.7 ± 1.2	4.5 ± 3.1	< 0.001
ALT (U/L)	29 ± 16	29 ± 9	0.908
Creatinine (mg/dL)	0.93 ± 0.12	0.92 ± 0.12	0.060

*Note:* Results are presented as mean ± standard deviation (SD). HOMA-B% = beta cell function; HOMA-S% = beta cell sensitivity; HbA1c; hemoglobin A1c or glycated hemoglobin.

Abbreviations: ALT, alanine transferase; GINR, glucose insulin ratio; HDL, high-density lipoproteins; HOMA-IR, homeostasis model of assessment insulin resistance; LDL, low-density lipoproteins; QUICKI, quantitative insulin sensitivity check index; Secretory-HOMA, secretory homeostasis model of assessment; TC: HDL-C, total cholesterol and HDL-cholesterol ratio.

**Table 3:** Frequency distribution of the *KCNJ11* genotype and risk of diabetes.

Genotype	Study subjects		OR (95% CI)	*p* value
	Diabetic (*n* = 371) (*n*, %)	Control (*n* = 326) (*n*, %)		
E23E	198 (53.37)	229 (70.25)	1 (reference)	—
E23K	159 (42.86)	91 (27.91)	2.02 (1.47–2.78)	< 0.001
K23K	14 (3.77)	6 (1.84)	2.70 (1.02–7.16)	< 0.05
E23K + K23K	173 (46.63)	97 (29.75)	2.06 (1.51–2.82)	< 0.001

*Allele (n, frequency)*				
E allele	277 (0.748)	274 (0.842)	1 (reference)	—
K allele	94 (0.252)	52 (0.158)	1.79 (1.23–2.61)	< 0.01

*Note:* Results are expressed as numbers (percentages). E23E, wild-type homozygote; E23K, heterozygote variants; K23K, homozygote variants.

**Table 4:** Frequency distribution of the *KCNJ11* (E23K) genotypes in the study subjects according to gender.

Gender	Genotype	Study subjects		OR (95% CI)	*p* value
		Diabetic (*n* = 371)	Control (*n* = 326)		
		*n* = 177 (*n*, %)	*n* = 172 (*n*, %)		
Male	E23E	93 (52.5)	114 (66.3)	1 (reference)	—
	E23K	76 (42.9)	53 (30.8)	1.75 (1.13–2.74)	< 0.05
	K23K	8 (4.5)	5 (2.9)	1.96 (0.62–6.19)	ns

		***n* = 194 (*n*, %)**	***n* = 154 (*n*, %)**		

Female	E23E	105 (54.1)	115 (74.6)	1 (reference)	—
	E23K	83 (42.8)	38 (24.7)	2.39 (1.50–3.81)	< 0.001
	K23K	6 (3.1)	1 (0.6)	6.57 (0.78–55.5)	ns

*Note:* Results are expressed as numbers (percentages). *KCNJ11* genotypes; E23E, wild-type homozygote; E23K, heterozygote variants; K23K, homozygote variants.

Abbreviation: ns = not significant.

**Table 5:** Frequency distribution of the *KCNJ11* genotype with risk of diabetes according to the family history of diabetes.

Family history of diabetes	Genotype	Study subjects		OR (95% CI)	*p* value
		Diabetic (*n* = 371)	Control (*n* = 326)		
		*n* = 258 (*n*, %)	*n* = 132 (*n*, %)		
Yes	E23E	132 (51.1)	96 (72.8)	1 (reference)	—
	E23K	116 (45.0)	32 (24.2)	2.64 (1.65–4.23)	< 0.001
	K23K	10 (3.9)	4 (3.0)	1.82 (0.55–5.97)	ns

		***n* = 113 (*n*, %)**	***n* = 194 (*n*, %)**		

No	E23E	66 (49.6)	133 (68.6)	1 (reference)	—
	E23K	43 (32.3)	59 (30.4)	1.47 (0.90–2.40)	ns
	K23K	4 (3.0)	2 (1.0)	4.03 (0.72–2.58)	ns

*Note:* Results are expressed as numbers (percentages). *KCNJ11* genotypes; E23E, wild-type homozygote; E23K, heterozygote variants; K23K, homozygote variants.

Abbreviation: ns = not significant.

**Table 6:** Multinomial logistic regression analysis for risk factors of diabetes with the *KCNJ11* genotype in subjects with T2DM.

Covariates	B		SE	*p* value	OR	95% CI
Gender	−0.356		0.292	0.222	1.428	0.81–2.53
BMI (kg/m^2^)	−0.114		0.040	< 0.01	1.121	1.04–1.21
Glucose (mM/L)	3.229		0.536	< 0.001	0.040	0.01–0.11
Insulin (μU/L)	0.036		0.109	0.738	0.964	0.78–1.19
HOMA-B%	0.020		0.008	0.009	0.980	0.97–1.00
HOMA-S%	0.004		0.010	0.663	0.996	0.98–1.02
HOMA-IR	0.090		0.984	0.927	0.914	0.13–6.28
Triglycerides (mg/dL)	−0.007		0.003	< 0.05	1.007	1.00–1.01
T cholesterol (mg/dL)	−0.012		0.004	< 0.01	1.012	1.00–1.02
HDL–C (mg/dL)	−0.085		0.015	< 0.001	1.089	1.06–1.12
*KCNJ11* (E23K)	−0.570		0.863	0.509	1.768	0.33–9.59
*KCNJ11* genotype (E23K + K23K)	1.745		0.985	0.076	0.175	0.03–1.21

*Covariates after adjusting for confounding factors*						
KCNJ11 (E23K)		−0.289	0.505	0.567	0.749	0.278–2.02
KCNJ11 genotype (E23K + K23K)		−0.414	0.563	0.462	0.661	0.219–1.99

*Note:* The reference category was E23E variants. Multinomial logistic regression analysis was adjusted with gender, BMI, glucose, insulin, HOMA-B%, HOMA-S%, HOMA-IR, triglycerides, total cholesterol, and HDL–cholesterol, respectively.

Abbreviation: ns = not significant.

## Data Availability

All relevant data are available at Dhaka University Institutional Repository as Ph.D. Thesis, URI: http://repository.library.du.ac.bd:8080/xmlui/xmlui/handle/123456789/1716 [31].
